# Seeing elements by visible-light digital camera

**DOI:** 10.1038/srep45472

**Published:** 2017-03-31

**Authors:** Wenyang Zhao, Kenji Sakurai

**Affiliations:** 1 University of Tsukuba, 1-1-1, Tennodai, Tsukuba, Ibaraki, 305-0006, Japan; 2 National Institute for Materials Science, 1-2-1, Sengen, Tsukuba, Ibaraki, 305-0047, Japan

## Abstract

A visible-light digital camera is used for taking ordinary photos, but with new operational procedures it can measure the photon energy in the X-ray wavelength region and therefore see chemical elements. This report describes how one can observe X-rays by means of such an ordinary camera - The front cover of the camera is replaced by an opaque X-ray window to block visible light and to allow X-rays to pass; the camera takes many snap shots (called single-photon-counting mode) to record every photon event individually; an integrated-filtering method is newly proposed to correctly retrieve the energy of photons from raw camera images. Finally, the retrieved X-ray energy-dispersive spectra show fine energy resolution and great accuracy in energy calibration, and therefore the visible-light digital camera can be applied to routine X-ray fluorescence measurement to analyze the element composition in unknown samples. In addition, the visible-light digital camera is promising in that it could serve as a position sensitive X-ray energy detector. It may become able to measure the element map or chemical diffusion in a multi-element system if it is fabricated with external X-ray optic devices. Owing to the camera’s low expense and fine pixel size, the present method will be widely applied to the analysis of chemical elements as well as imaging.

Generally a visible-light digital camera is used for taking photos in visible light, however, it can have a new function to measure the photon energy of X-rays. The new function is based on the basic working principle of digital cameras. When a photon hits a pixel photodiode on the camera sensor chip, charges are generated. The amount of charges is proportional to the photon energy^1^. In the case of visible light, one photon can only generate one charge, therefore, a digital camera cannot distinguish the photon energy or the color of visible light. In the case of X-rays, one photon can generate thousands of charges as its photon energy is thousands of times higher than visible light. The quantity of charges is recorded in the form of pixel intensity and then the corresponding photon energy is implied. If the X-rays are fluorescent X-rays emitted from atoms, the corresponding elements can be identified by matching their characteristic fluorescence energy. In short, a visible-light digital camera can be applied to identify chemical elements.

In order to individually measure each photon’s energy, the recorded photon events on one camera image should be dispersed sparsely to separate them. This special condition is generally named as single photon counting mode. It is a prerequisite when applying a camera to X-ray spectroscopy measurement. Single photon counting mode can be easily achieved by reducing the amount of X-ray photon events on each image. Related discussion dates back to at least the 1980s^2^, and from then on it was applied to both special X-ray charge-coupled-device (CCD) cameras^3^,^4^,^5^ and conventional CCD cameras^6^,^7^,^8^,^9^,^10^. Nevertheless, none of these CCD cameras are frequently seen as current ordinary visible-light digital cameras. On the other hand, these days complementary-metal-oxide-semiconductor (CMOS) cameras are becoming more and more popular for both domestic appliances and scientific research. CMOS cameras are also used for X-ray imaging^11^,^12^,^13^,^14^, however, they are rarely used in direct X-rays. Scintillator layers are always installed in front of the image sensor to convert X-rays to visible light. In this case, information on X-ray photon energy is frequently lost. In this research, we use a visible-light CMOS camera for direct X-ray measurement. The ordinary digital camera becomes able to measure X-ray energy-dispersive spectra when it is specially operated and the camera images are properly processed.

In order to obtain high quality X-ray spectra, the adopted camera should be of high quality as well. In most cases, the larger the better for the camera’s dynamic range and the readout noise should be as small as possible. In this research, we use a commercially-available scientific CMOS (sCMOS) camera^15^,^16^. Its analog to digital (A/D) conversion is 16 bits. It is named as “scientific” because it is suitable for scientific research because of its high quality and reliable performance. As the camera is designed to take photos in visible light, its front cover is made of transparent glass and an optical lens system is installed in front of the cover. As the transparent glass cover is 2 mm thick, X-rays cannot go through it. In order to fit out the camera for direct X-ray measurement, the lens system and the glass cover are removed to allow X-rays to pass, and an opaque front cover with an X-ray window is installed to avoid the influence of visible light. Detailed information on the sCMOS camera and cover replacement procedures are described in the method section.

As stated earlier, the camera should work in single photon counting mode to measure X-ray energy-dispersive spectra. The amount of photon events on one camera image should be carefully controlled by adjusting the intensity of incident X-rays or adjusting the single exposure time. When the camera image does not show any continuous pattern but only shows many discrete dots, the condition of single photon counting mode is thought to be achieved. The next step is to process the camera images. Generally the X-ray photon energy can be retrieved from the pixel intensities of the corresponding photon event on the image, however, the retrieval process is not always straightforward. In this research, we propose an integrated-filtering method to ensure the retrieval process is effective and correct, then high quality X-ray fluorescence (XRF) spectra can be readily obtained after processing the raw camera images.

In this report, we demonstrate the entire procedure for using a visible-light digital camera for X-ray spectroscopy measurement. Some key properties such as energy resolution and count rate saturation are tested and discussed. It is shown that the camera can successfully analyze the composition of elements in unknown samples. The great potential of applying such visible-light cameras to X-ray detection is also explained.

## Results

### Retrieving X-ray energy-dispersive spectra from camera images

Prior to the experiment, 100 dark images are acquired without any illumination of visible light or X-rays. It is found that the average intensity of an unilluminated pixel is approximately 100. In different images, the intensity of the same pixel may vibrate around 100 because of the dark current. The frequency distribution of all pixel intensities in all the dark images shows a symmetric sharp peak at 100. The full width at half maximum (FWHM) of this background peak is 8, implying the level of dark current. An average background image is generated by averaging all the dark images. As the pixels on the sCMOS sensor are quite independent of each other, the exact average intensity of different pixels is not totally the same, hence the average background image should be subtracted from every experimental image to eliminate the fixed-pattern noise. Generally speaking, the level of dark current is not influenced by external X-rays.

In the experiment, the primary X-ray beam comes from a laboratory X-ray tube of copper target. A crystal monochromator is employed to obtain monochromatic copper Kα X-rays, hence copper Kβ X-rays the continuous Bremsstrahlung radiation background of the primary X-ray beam can be eliminated. A sample containing vanadium pentoxide powders is illuminated by the primary X-rays. The camera is placed in front of the sample to receive the fluorescent and scattering X-rays. The single exposure time is set as 100 ms to satisfy the requirements for single photon counting mode. The single exposure is repeated for 6,000 times, hence 6,000 independent images are acquired to accumulate sufficient signals.

**Figure 1:**
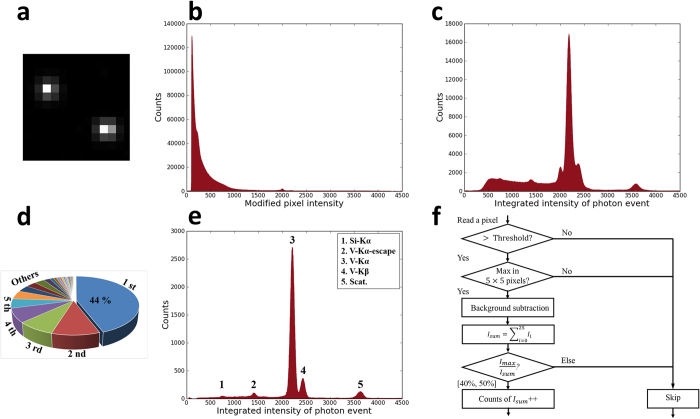
Principle of retrieving X-ray energy-dispersive spectra from camera images. (**a**) shows a typical part of one image acquired by the sCMOS camera. (**b**) shows the frequency distribution of pixel intensities after subtracting the average background image. (**c**) shows the frequency distribution of the 5 × 5 integrated intensities of all photon events. (**d**) is a conceptual drawing of the [40%, 50%] filtering criteria. (**e**) shows the final X-ray fluorescence spectra obtained after filtering. (**f**) is the flow chart of the overall procedures for data analysis.

Figure 1a shows a typical part of one camera image. It has two discrete bright dots. The pixels in black background are not influenced by X-ray photons, so their intensities are around 100. The intensity of the white pixels in the two dots is obviously higher than the background value of 100, generally from hundreds to thousands. The frequency distribution of pixel intensities in all 6,000 images is counted. In order not to count the huge amount of unilluminated pixels, only pixels whose intensity is larger than 200 are considered. After that, their intensities are respectively modified by subtracting the average background image. The final frequency distribution histogram is shown in Fig. 1b. The frequency continuously decreases from the low intensity side to the high intensity side. A very small peak appears at around 2,000. Obviously, this frequency distribution histogram doesn’t match the XRF spectra of the sample at all.

As the bright pixels are always gathered in discrete bright dots, it is reasonable to infer that the pixels in one bright dot are illuminated by the same X-ray photon and one bright dot corresponds to one photon event. That is to say, the charges generated by an X-ray photon are shared by a group of neighboring pixels. This phenomenon of charge sharing is frequently seen in many CCD cameras as well^1^,^5^,^10^,^17^. Unlike a visible-light photon, an X-ray photon can generate a charge cloud of thousands of photoelectrons. The charge cloud has a certain size in the photodiode matrix of the image sensor, so that the charges will be collected by either a single pixel or some neighboring pixels. In previous reports on CCD cameras, there are both single-pixel photon events and multi-pixel photon events. The size of a multi-pixel photon event is generally smaller than 3 × 3 pixels. Nevertheless, in our research on this sCMOS camera, nearly all photon events are multi-pixel events. The size of a multi-pixel event generally exceeds the region of 3 × 3 pixels. These differences are probably due to different pixel sizes and anatomies of the CCD sensor and sCMOS sensor. The minimum CCD pixel size in previous related works is 13 μm × 13 μm^10^, and it is only 6.5 μm × 6.5 μm in this sCMOS camera. As a consequence, it is natural that more pixels are needed to collect one charge cloud.

A threshold of 200 is proposed to pick out photon events from the image background. It has been found that the pixel intensities in dark images can hardly exceed 200. Based on the study of the 100 dark images, the probability is about 50 ppm. As a consequence, if the intensity of a pixel is higher than 200 in an X-ray image, a photon event can be recognized at this position. The next step is to collect all signal pixels of this photon event. As shown in Fig. 1a, the white pixels in bright dots are obviously signal pixels because their intensities are very high. However, arriving at a judgment on the grey pixels surrounding the bright dots is not straightforward. Sometimes their intensities are only slightly higher than the background value of 100, so that it is difficult to determine whether their intensities are from dark current or X-ray signals. In order to fully collect all signal pixels, all pixels in the region of 5 × 5 pixels are considered. The center of the 5 × 5 region should be the pixel whose intensity is the maximum in the photon event. It is recognized as the impinging position of the X-ray photon. In most cases, the region of 5 × 5 pixels is large enough to include all signal pixels in one photon event. After subtracting the average background image, the intensities of all 25 pixels are integrated and the frequency distribution of the integrated pixel intensity is counted. This frequency distribution histogram is shown in Fig. 1c. This time, the expected XRF spectra of the sample appears preliminarily. However, a strong background in the low energy side also exists.

The background in the low energy side comes from the photon events of incomplete charge collection. As the sCMOS camera is designed for visible light, its depletion region of photodiodes is not thick enough to fully absorb all high energy X-ray photons. Some X-ray photons may penetrate the depletion region and be absorbed in field-free regions below. The charges generated in the field-free regions diffuse rapidly. Some of the charges can still be collected if they spread to the depletion region, whereas the rest of the charges are lost. As a result, the integrated intensity of a photon event may underestimate the true X-ray photon energy. That is why most of the background appears in the low energy side. There have been some similar discussions on thin-depletion-region CCD cameras^17^. We think such discussions can be generalized to include sCMOS cameras because the basic photodiode principle is not very different.

It is necessary to distinguish and remove all incomplete-charge-collection photon events. If an X-ray photon is absorbed in the depletion region, the generated charges will be quickly collected with no loss. In this case, the size of the charge cloud should not be very large and the center pixel should collect many more charges than other pixels. In contrast, if the X-ray photon is absorbed in field-free regions, the charge cloud diffuses rapidly, and then only a portion of the charges can be collected. In this case, the ratio of charges collected by the center pixel to all collected charges cannot be distinctly high. For this reason, the center-to-integrated ratio can serve as an indicator to identify incomplete-charge-collection photon events. In order to determine the ratio criteria, the center-to-integrated ratios of photon events in different regions of the spectra are surveyed. We define two regions of interest (ROI) in Fig. 1c. One ROI is in the main peak at around 2200, which is expected to be the strong Kα peak of vanadium in the sample; the other ROI is in the low energy background from 500 to 2000, which is thought to be made of incomplete-charge-collection photon events. The frequency distributions of the center-to-integrated ratio in the two ROIs are counted respectively. It has been found that the center-to-integrated ratios of nearly all photon events in the background ROI are below 40%, whereas the ratios of some photon events in the peak ROI can exceed 40%. As a consequence, the percentage of 40% is decided on as the empirical criteria to filter out incomplete-charge-collection photon events.

After applying the filtering criteria of 40%, most of the low energy background is removed. However, there are some unknown peaks left in the low energy side. In addition, the expected fluorescence peaks are not exactly symmetric and the high-energy extensions are much stronger. In order to trim the peaks to make them symmetric, the main peak in Fig. 1c is divided into different ROIs and the center-to-integrated ratios of the different ROIs are surveyed again. This time it is found that the high-energy extensions of fluorescence peaks as well as the unknown noisy peaks in the low energy side can be removed by filtering out the photon events whose center-to-integrated ratio is larger than 50%. The physical reason for this criteria of 50% is still under investigation.

To sum up, a filtering criteria of [40%, 50%] is determined. As shown in Fig. 1d, only when the center-to-integrated ratio of one photon event is in [40%, 50%] can it be counted as a valid signal. This filtering criteria may filter out 90% of photon events. However, the remaining photon events can make a perfect XRF spectra (Fig. 1e). The scattering peak of primary copper Kα and the fluorescence peak of vanadium Kα and Kβ can be recognized. As the camera sensor is Si-based, the small silicon Kα peak and the corresponding escape peak of vanadium Kα are also shown. In the meantime, small adjustments to the numerals in this criteria such as [39%, 51%] will not make the final spectra very different. However, we prefer to fix it as [40%, 50%] after comparing the final spectra’s quality and signal to background ratio. The detailed procedures for finding these empirical criteria are given in the supplementary materials.

It should be noted that, the filtering criteria of [40%, 50%] has been discovered from our surveys using a variety of known samples. This method works very well, and it is highly reliable. It has been found that the same filtering criteria always leads to correct XRF spectra with no risk of subjective bias, whatever the sample is. The main idea is that the center-to-integrated ratio correlates with the spread and the shape of the charge distribution. For example, for the Gaussian distribution with standard deviation of σ, the central area covering upto 0.5–0.7σ corresponds to the 40–50% intensity ratio. The present method uses such relationship to filter out inappropriate distribution as an invalid event.

Figure 1f summarizes the flow chart of the overall integrated-filtering method for analyzing camera images. All pixels in camera images except those in borders are examined by this flow chart to retrieve the X-ray energy-dispersive spectra measurement. We have coded the integrated-filtering method in the camera controlling software, therefore the X-ray spectra can be output immediately after measurement.

**Figure 2:**
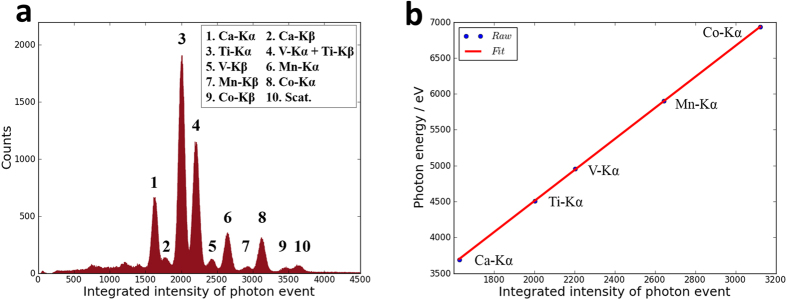
Calibration from the integrated pixel intensity of photon event to X-ray photon energy. (**a**) is the X-ray fluorescence spectra of the standard sample containing calcium, titanium, vanadium, manganese and cobalt. The five main Kα peaks in the spectra are taken as the reference peaks and paired with their respective photon energy. The linear fitting result is shown in (**b**).

In order to measure unknown samples, the x-axis of the acquired XRF spectra should be calibrated from integrated intensity to X-ray photon energy. Because the integrated pixel intensity, the amount of charges and the X-ray photon energy are proportional to each other, the calibration function should be linear. Generally the calibration is conducted by measuring a standard sample. The standard sample contains calcium, titanium, vanadium, manganese and cobalt. As shown in Fig. 2a, the fluorescence peaks from calcium Kα to cobalt Kβ are all recognized. The integrated intensities of the five strong Kα peaks are paired with their respective photon energy. The linear fitting result is shown in Fig. 2b. The root-mean-square-error (RMSE) of these five data points is 2.3 eV. The small error shows that the XRF spectra obtained by the sCMOS camera are accurate enough to identify unknown peaks and unknown elements.

### Energy resolution in X-ray energy-dispersive spectra

**Figure 3:**
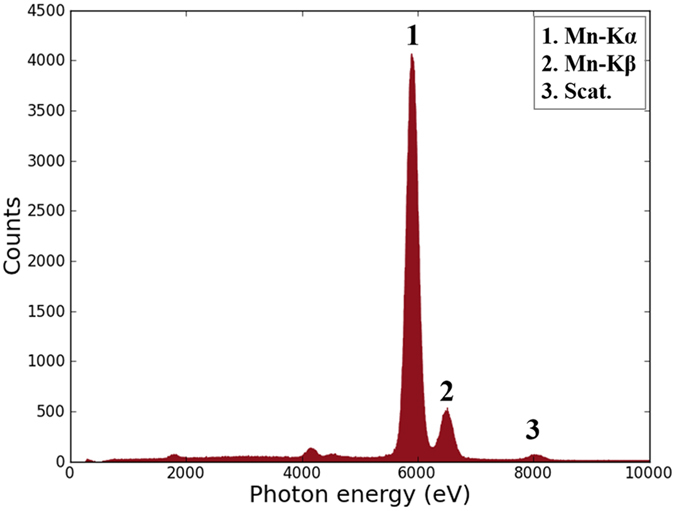
X-ray fluorescence spectra of MnO_2_ obtained by the sCMOS camera. The energy resolution is 220 eV by checking the full-width-at-half-maximum of the Mn Kα peak.

The energy resolution of the camera is tested by measuring a sample containing manganese dioxide powders. The sample is excited by monochromatic copper Kα X-rays. The XRF spectra are shown in Fig. 3. By checking the FWHM of the manganese Kα peak, the energy resolution is determined as 220 eV at 5898 eV (manganese Kα).

### Saturation of count rates

**Figure 4:**
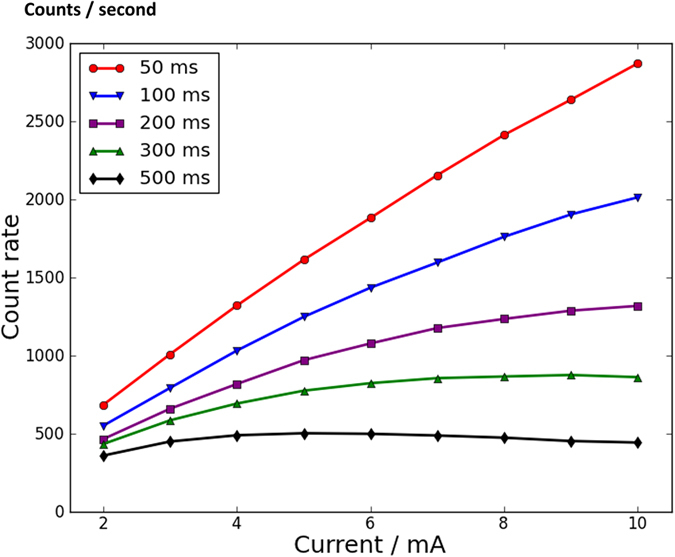
Count rates of vanadium fluorescence Kα signals detected by the sCMOS camera. The intensity of X-rays is proportional to the tube current. When the time of single exposure becomes shorter and the corresponding frame rate becomes quicker, the count rate capacity becomes larger. When the single exposure time is 50 ms, the function of the count rates to the tube current is linear, indicating that the count rate capacity is sufficient to measure such normal intensity X-rays.

In single photon counting mode, the amount of incident X-ray photons in single exposure time should be strictly controlled to avoid the overlap of two or more photon events in one image. Therefore, it is generally thought that single photon counting mode can only work with low intensity X-rays and the camera is easily saturated with high intensity X-rays. However, when the time of a single exposure becomes shorter and the frame rate becomes quicker, the count rate capacity of the camera is able to become larger to catch higher intensity X-rays. If the frame rate of the camera is quick enough, the camera’s count rate capacity can become large enough to measure normal intensity X-rays rather than simply low-intensity X-rays. One experiment is conducted to confirm this. The experiment conditions are quite similar to the conditions for Fig. 1. The sample is the same sample which contains vanadium pentoxide powders. The primary beam is monochromatic copper Kα X-rays from a sealed type copper X-ray tube. The tube voltage of the X-ray source is fixed at 20 kV, thus the intensity of fluorescent X-rays increases proportionally as the tube current increases. The camera is able to acquire images continuously, so that the frame rate is an inverse of the single exposure time. In different conditions of single exposure time, the detected count rates of vanadium fluorescence Kα photons are plotted with respect to tube current (Fig. 4). It can be noted that the saturation of count rate comes quite early when a long single exposure time such as 500 ms is set. After saturation, the count rate decreases. This is because there are too many photon events in one image so that some of them may be overlapped and then filtered out by the criteria of center-to-integrated ratio. In contrast, the saturation of count rates becomes larger when the length of a single exposure becomes shorter. When the single exposure time is set as 50 ms, the function of the count rates to the tube current is nearly linear, indicating that the capacity of count rates is abundant enough to measure the high-intensity X-rays in this experiment and there is no risk of saturation.

In routine experiments, the parameter of single exposure time should be determined based on practical experiment conditions. A short single exposure time should be used with high-intensity X-rays to avoid saturation, while a long single exposure time is also acceptable with low-intensity X-rays.

### Application of distinguishing chemical elements

**Figure 5:**
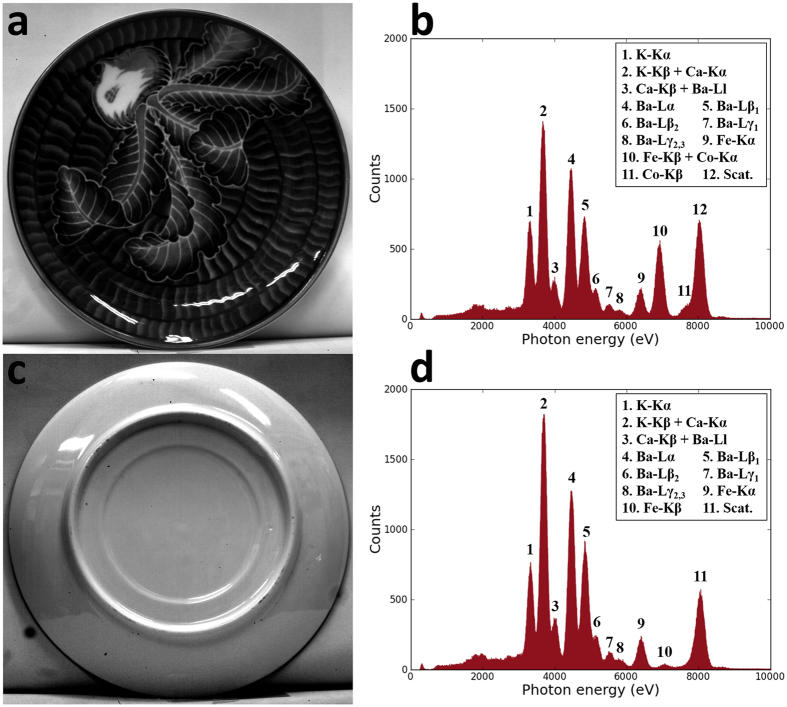
Photos and X-ray fluorescence spectra of a ceramic plate obtained by the same sCMOS camera. (**a**) is the photo of the ceramic plate’s front side and (**b**) is its X-ray fluorescence spectra; (**c**) is the photo of the back side and (**d**) is its spectra. The photos and the X-ray fluorescence spectra are obtained by the same sCMOS camera.

The visible-light digital camera can measure XRF spectra of samples, and therefore the composition of elements in the sample can be analyzed as well. For example, a ceramic plate which has a white base (Fig. 5c, back side) and blue patterns (Fig. 5a, front side) is tested. The two photos in Fig. 5a and c are taken by the sCMOS camera before removing the optical lens system and the transparent glass cover. The photos are monochrome as there is no color filter in the camera. After removing the lens system and replacing the glass cover with an X-ray window, the XRF spectra of the front side (Fig. 5b) and back side (Fig. 5d) are respectively measured by the same sCMOS camera. In the experiment, the primary X-ray beam is monochromatic copper Kα X-rays. The exposure time for one single image is 100 ms. The accumulation time is 30 minutes. In the spectra, every peak can be identified, thus the corresponding elements can be identified as well. It could be noted that the element composition of the front side and the back side are quite similar, whereas cobalt only appears in the front side, indicating the relations between cobalt and the blue color of the ceramic.

In XRF analysis, the sCMOS camera is used in the same way as other existing X-ray detectors. Therefore, it is possible to be used for quantitive XRF analysis to determine the concentration of elements in the sample by either the use of an experimentally obtained calibration curve or so-called reference-free analysis based on X-ray fundamental parameters. It is also worthy of mentioning that the use of sCMOS camera is suitable for the analysis of trace elements particularly when combined with total-reflection geometry.

## Discussions

Conventional visible-light digital cameras are developed for taking ordinary photos. As a kind of visible-light camera, an sCMOS camera is of very high quality so that it has been widely introduced in many areas of scientific research for recording visible light signals^18^,^19^. There are also some reports on using sCMOS cameras for X-ray imaging. However, scintillator layers are always needed to convert X-rays to visible light, and it is very rare to expose a bare sCMOS sensor to direct X-rays. Obtaining the spectroscopy information of X-rays is generally difficult for sCMOS cameras. In this research, it has been found that an ordinary sCMOS camera can work with direct X-rays and measure X-ray photon energy. The operational procedures include replacing the front cover, using the camera in single photon counting mode and analyzing camera images by the integrated-filtering method. All these operations do not require any modifications to the inner structures or on-chip electronics of the sCMOS camera and they can be conducted smoothly. In conclusion, the XRF spectra obtained by the sCMOS camera have highly accurate channel-to-energy calibration and a fine energy resolution of 220 eV. The count rate capacity is enough acceptable for routine laboratory XRF experiments. As a consequence, the visible-light sCMOS camera could be easily introduced to routine XRF analysis as a spectrometer. The composition of elements in samples can be analyzed as well.

In the measurement of X-ray photon energy, sCMOS cameras and traditional semiconductor detectors such as silicon drift detectors (SDD)^20^,^21^ have similar working mechanisms. Both of them are quantifying the amount of charges generated by an X-ray photon by amplifiers and analog-to-digital convertors. The major difference is that, in SDD, all charges drift to and are collected by one electrode, whereas in sCMOS camera the charges diffuse to and are collected by many pixels. Meanwhile, the reasons for count rate saturation are also similar. When one photon event is inseparable from other events, it is called a piling-up photon event and it should be rejected. The probability of rejected pile-up photon events increases as the intensity of X-rays increases. In the case of SDD, the pile-up phenomenon of photon events happens in the temporal dimension^22^; while in the case of sCMOS camera, the pile-up phenomenon of photon events happens in the same camera image.

At present the sCMOS camera is not so competitive as SDD in spectra analysis. For example, the energy resolution of SDD can reach around 130 eV at manganese Kα^21^ while that of sCMOS camera is 220 eV in this research; the count rates of an ordinary SDD can be more than 500,000 or even several million counts per second and that of the sCMOS camera is only several thousand. However, the sCMOS camera has its unique advantages. The size of its active area is 16.6 mm × 14 mm, approximately 230 mm^2^, which is extremely difficult to achieve by a single SDD. Therefore sCMOS camera is more suitable for X-ray spectroscopy experiments in which a large cross section or large solid angle of the detector are required. In addition, as a single SDD has no position sensitivity, it cannot record the position information of X-ray photons. Some multi-cell SDDs^23^ are developed for position sensitive XRF analysis. However, their pixel amount is limited compared to the sCMOS camera which has more than 5 million pixels, and therefore they cannot really serve as 2-dimensional X-ray energy detectors.

There are other special X-ray imagers such as ePix100^24^, Pilatus^25^, Eiger^26^ and pnCCD^4^. Some of them may simultaneously possess both position sensitivity and spectroscopy properties. Generally speaking they have high X-ray detection efficiency and high count rates, whereas their pixel amount and spatial resolution are limited by manufacturing technique or expense. Frequently the pixel amount is in the tens of thousands and the pixel size is in the several tens of microns. Neither of them is as good as the sCMOS camera. On the other hand, the comparison between conventional CCD cameras with the sCMOS camera can be more interesting. A conventional CCD camera can also work as an X-ray spectrometry imager when it works in single photon counting mode^10^,^27^. The adopted CCD camera possesses many advanced features so that it is more like a professional detector rather than an ordinary visible-light digital camera. Its energy resolution can reach 133 eV at manganese Kα in the best condition, while the energy resolution of the sCMOS camera is 220 eV in this research. During measurement, the CCD camera is cooled to below − 85 °C while the sCMOS camera works in a more tolerant condition of 5 °C. This is inferred to be one of the reasons for different energy resolution, because the level of dark current may influence the energy resolution and it is proportional to the working temperature. The sCMOS camera offers more pixels and smaller pixel size (2560 × 2160 pixels, pixel size 6.5 μm × 6.5 μm) than the CCD camera (1024 × 1024 pixels, pixel size 13 μm × 13 μm). In addition, as a kind of active pixel sensor (APS), every pixel on the sensor chip of sCMOS cameras has an amplifier and can be controlled independently, therefore, the sCMOS camera does not need to transfer charges from pixel to pixel. As a consequence, the sCMOS camera can be free of the smearing effect which may contaminate the XRF spectra obtained by the CCD camera^27^, and it can achieve a high frame rate more easily.

In the future, the sCMOS camera is a promising candidate for full filed XRF imaging by installing external X-ray optics such as a micro pinhole^9^,^10^ or a capillary plate^27^,^28^,^29^,^30^,^31^, and thus the element distribution in samples can also be measured. Previously the detector used for full filed XRF imaging has been either a professional CCD camera or a special X-ray detector like pnCCD. Now the sCMOS camera can become a new option with competent energy resolution, acceptable count rates, larger pixel amount, smaller pixel size and low expense. As it is just an ordinary visible-light digital camera and it can be easily obtained in the market, it should be capable of promoting the popularization of the full field XRF imaging technique and make the detection of element spatial distribution easier.

## Methods

### Preparation of the visible-light digital camera

The camera used in this research is a PCO.edge 5.5 sCMOS camera from PCO AG^16^. The specifications of this sCMOS camera are shown in Table 1. In the experiment, the sCMOS camera uses the rolling shutter mode to read camera images, which means the pixel reset and exposure restart is carried out row by row. Therefore, image exposure and image reading can proceed simultaneously. The image acquisition is continuous with no interruption if the exposure time is not shorter than the frame reading time. In this research, the pixel clock is always set as slow mode to ensure the image quality. In this condition, the maximum frame rate can reach 33 frames per second. The detailed difference between quick mode and slow mode has not been compared as yet. During measurement, the function of B/W noise filter should be switched off to avoid artificial disturbance.

In order to fit the camera to direct X-ray measurement, the optical lens system in front of the camera is removed. The front cover is a 2-mm-thick visible-light transparent glass, and it is replaced with a 2-mm-thick aluminum alloy plate. On the plate, there is a circular aperture with a diameter of 4 mm. An X-ray window is pasted on the aperture. The X-ray window employed is commercially available 12 micron thick polymer film coated on both sides with 0.2 micron thick metallic aluminum (Ube Industries, Ltd.). To reduce the light leakage caused by small pinholes (unsuccessfully coated parts), we layered two (sometimes three) sheets of the film. The film is strong enough mechanically and stable. The aluminum coating blocks visible light, but is almost transparent for X-rays. The use of much thinner X-ray window is promising to analyze lower energy X-ray fluorescence spectra, though the present experiments were done for rather high energy. The distance between the X-ray window and the sCMOS sensor is approximately 6 mm, therefore, the illumination area on the sCMOS sensor is much larger than the size of the X-ray window when the sCMOS camera is close to samples in XRF experiment. However, the 4 mm dia. aperture more or less contributes to reduce unnecessary X-ray photons. If a small pinhole (such as 10–50 micron dia) is placed at the center of this aperture, the same present system can work as an imager of the elements. For ordinary X-ray fluorescence spectroscopy to see elements, we do not need to use such a pinhole.

The replacement of the camera cover was done in a glove box filled with nitrogen gas.

The photos for replacing the front covers are given in the supplementary materials.

## Additional Information

**How to cite this article**: Zhao, W. and Sakurai, K. Seeing elements by visible-light digital camera. *Sci. Rep.* **7**, 45472; doi: 10.1038/srep45472 (2017).

**Publisher's note:** Springer Nature remains neutral with regard to jurisdictional claims in published maps and institutional affiliations.

## Supplementary Material

Supplementary Information

## Figures and Tables

**Table 1:**
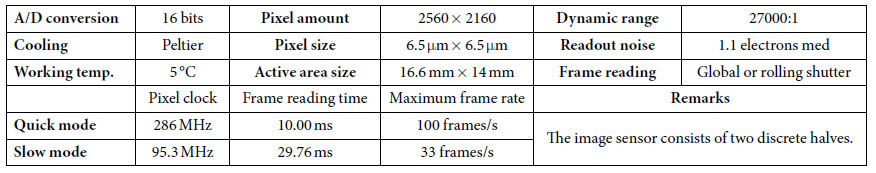
Specifications of the sCMOS camera.

## References

[b1] Tsunemi H., Hiraga J., Yoshita K., Miyata E. & Ohtani M. Comparison of methods of measuring the primary charge-cloud shape produced by an X-ray photon inside the CCD. Nucl. Instruments Methods Phys. Res. Sect. A Accel. Spectrometers, Detect. Assoc. Equip. 439, 592–600 (2000).

[b2] Griffiths R. E., Polucci G., Mak A., Murray S. S. & Schwartz D. A. Preliminary Results From A Single-Photon Imaging X-ray Charge Coupled Device (CCD) Camera. Proc. SPIE 244, 57 (1981).

[b3] Strüder L., Holl P., Lutz G. & Kemmer J. Development of fully depletable CCDs for high energy physics applications. Nucl. Instruments Methods Phys. Res. Sect. A Accel. Spectrometers, Detect. Assoc. Equip. 257, 594–602 (1987).

[b4] Strüder L. . The European Photon Imaging Camera on XMM-Newton: The pn-CCD camera. Astron. Astrophys. 365, L18–L26 (2001).

[b5] Scharf O. . Compact pnCCD-based X-ray camera with high spatial and energy resolution: A color X-ray camera. Anal. Chem. 83, 2532–2538 (2011). 21355541 10.1021/ac102811p

[b6] Lumb D. H. Calibration and X-ray spectroscopy with silicon CCDs. Nucl. Inst. Methods Phys. Res. A 290, 559–564 (1990).

[b7] Tsunemi H., Wada M., Hayashida K. & Kwai S. X-ray color movie using the charge-coupled device with a direct x-ray detection method. Jpn. J. Appl. Phys. 30, 3540–3544 (1991).

[b8] Labate L. . Novel x-ray multispectral imaging of ultraintense laser plasmas by a single-photon charge coupled device based pinhole camera. Rev. Sci. Instrum. 78, 103506 (2007). 17979418 10.1063/1.2800774

[b9] Vasin M. G., Ignatiev Y. V., Lakhtikov A. E., Morovov A. P. & Nazarov V. V. Energy-resolved X-ray imaging. Spectrochim. Acta - Part B At. Spectrosc. 62, 648–653 (2007).

[b10] Romano F. P. . Macro and Micro Full Field X-Ray Fluorescence with an X-Ray Pinhole Camera Presenting High Energy and High Spatial Resolution. Anal. Chem. 86, 10892–10899 (2014). 25284509 10.1021/ac503263h

[b11] Rocha J. G., Ramos N. F., Lanceros-Mendez S., Wolffenbuttel R. F. & Correia J. H. CMOS X-rays detector array based on scintillating light guides. Sensors Actuators, A Phys. 110, 119–123 (2004).

[b12] Mokso R. . Advantages of phase retrieval for fast x-ray tomographic microscopy. J. Phys. D. Appl. Phys. 46, 494004 (2013).

[b13] Hall C. . Detectors for the Imaging and Medical Beam Line at the Australian Synchrotron. J. Instrum. 8, C06011–C06011 (2013).

[b14] Zenyuk I. V., Parkinson D. Y., Hwang G. & Weber A. Z. Probing water distribution in compressed fuel-cell gas-diffusion layers using X-ray computed tomography. Electrochem. commun. 53, 24–28 (2015).

[b15] Fowler B. . A 5.5 Mpixel 100 frames/sec wide dynamic range low noise CMOS image sensor for scientific applications. Proc. SPIE 7536, 753607–753612 (2010).

[b16] https://www.pco.de/fileadmin/user_upload/pco-product_sheets/pco.edge_55_data_sheet.pdf.

[b17] Lumb D. H. & Holland A. D. Event recognition techniques in CCD X-ray detectors for astronomy. Nucl. Inst. Methods Phys. Res. A 273, 696–700 (1988).

[b18] Huang F. . Video-rate nanoscopy using sCMOS camera-specific single-molecule localization algorithms. Nat. Methods 10, 653–8 (2013). 23708387 10.1038/nmeth.2488 PMC3696415

[b19] Chen W. T. . High-Efficiency Broadband Meta-Hologram with Polarization- Controlled Dual Images (2014). 10.1021/nl403811d 24329425

[b20] Lechner P. . Silicon Drift Detectors for high count rate X-ray spectroscopy at room temperature. Nucl. Instruments Methods Phys. Res. Sect. A Accel. Spectrometers, Detect. Assoc. Equip. 458, 281–287 (2001).

[b21] Lechner P., Pahlke A. & Soltau H. Novel high-resolution silicon drift detectors. X-Ray Spectrom. 33, 256–261 (2004).

[b22] Beckhoff B., Kanngietzer B., Langhoff N., Wedell R. & Wolff H. Handbook of Practical X-Ray Fluorescence Analysis (Springer Science & Business Media, 2007).

[b23] Gaskin J. a. . Development of a silicon drift detector array: An x-ray fluorescnece spectrometer for remote surface mapping. 7441, 744118 744118–12 (2009).

[b24] Blaj G. . Future of ePix detectors for high repetition rate FELs. AIP Conf. Proc. 1741 (2016).

[b25] Henrich B. . PILATUS: A single photon counting pixel detector for X-ray applications. Nucl. Instruments Methods Phys. Res. Sect. A Accel. Spectrometers, Detect. Assoc. Equip. 607, 247–249 (2009).

[b26] Dinapoli R. . EIGER: Next generation single photon counting detector for X-ray applications. Nucl. Instruments Methods Phys. Res. Sect. A Accel. Spectrometers, Detect. Assoc. Equip. 650, 79–83 (2011).

[b27] Romano F. P. . Micro X-ray Fluorescence Imaging in a Tabletop Full Field-X-ray Fluorescence Instrument and in a Full Field-Particle Induced X-ray Emission End Station. Anal. Chem. 88, 9873–9880 (2016). 10.1021/acs.analchem.6b02811 27656755

[b28] Wroblewski T. An X-ray camera: Synchrotron Radiat. News 9, 14–19 (1996).

[b29] Sakurai K. Total-reflection X-ray fluorescence imaging: Spectrochim. Acta B 54, 1497–1503 (1999).

[b30] Sakurai K. & Eba H. Micro X-ray fluorescence imaging without scans: Toward an element-selective movie. Anal. Chem. 75, 355–359 (2003). 12553774 10.1021/ac025793h

[b31] Eba H., Ooyama H. & Sakurai K. Combination of projection-based XRF, XAFS and XRD imagings for rapid spatial distribution analysis of a heterogeneous material. J. Anal. At. Spectrom. 31, 1105–1111 (2016).

