# Supplementary material for: Seeing elements by visible-light digital camera
Source: Sci Rep. 2017 Mar 31;7:45472. doi: 10.1038/srep45472 (PMC5374443; doi:10.1038/srep45472)
Supplement: Supplementary Information [file srep45472-s1.pdf]

Supplementary Information

Seeing elements by visible-light digital camera

Wenyang Zhao[1,2] and Kenji Sakurai[2,1,a)]

[1]*University of Tsukuba, 1-1-1, Tennodai, Tsukuba, Ibaraki, 305-0006, Japan*

[2]*National Institute for Material Science, 1-2-1, Sengen, Tsukuba, Ibaraki, 305-0047, Japan*

a) Corresponding Author, sakurai@yuhgiri.nims.go.jp

S1. Modifications of the visible-light digital camera

The camera used in the research is the PCO.edge 5.5 sCMOS camera (Figure S1.1) from the PCO AG. It is designed to take photos in visible light. In the beginning, an optical lens system is installed on the sCMOS camera via a C-mount ring. The size of the sCMOS camera is 70 mm × 76 mm × 88.3 mm.

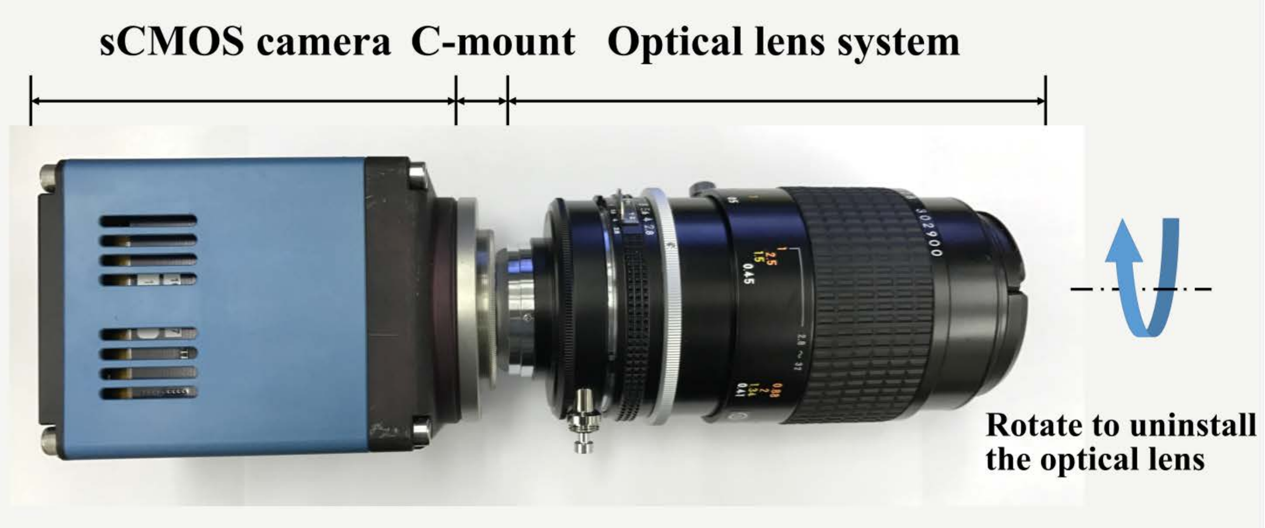

Figure S1.1 The PCO.edge 5.5 sCMOS camera from the PCO AG

We slightly modified the camera to fit it to direct X-ray measurement. Firstly, we uninstalled the optical lens system and unscrewed the C-mount ring (Figure S1.2). Then we could see the sCMOS camera's front cover, which is a 2-mm-thick transparent glass plate, and the sCMOS image sensor below the glass plate (Figure S1.3). Secondly, we replaced the transparent glass plate with an aluminum alloy plate of the same size. In the center of the plate, we made a circular aperture of which diameter is 4 mm (Figure S1.4). The aperture is used as an opening for receiving X-rays. A thin X-ray window is pasted on the hole to block visible light. The X-ray window employed is commercially available 12 micron thick polymer film of which both sides are coated by metallic

aluminum of 0.2 micron thick each (Ube Industries, Ltd.). To reduce the light leakage caused by small pinholes (unsuccessfully coated parts), we layered two (sometimes three) sheets of the film. The films are mechanically strong enough and stable. The aluminum coating blocks visible light, but is almost transparent for X-rays. Figure S1.5 shows the direct comparison of the sCMOS camera before and after cover replacement. The sCMOS camera after replacement displayed here is a same-type model. The replacement of the camera cover was done in a glove box filled with pure dried nitrogen gas.

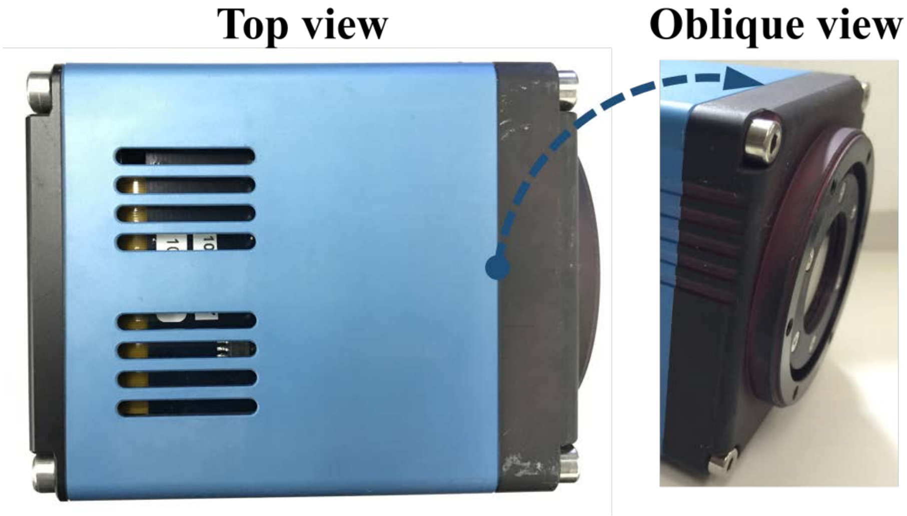

Figure S1.2. The sCMOS camera after removing the optical lens system and the C-mount

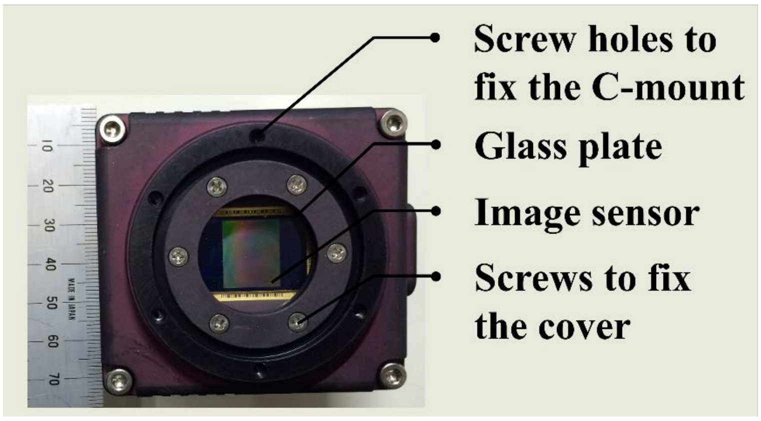

Figure S1.3 The front cover of the sCMOS camera

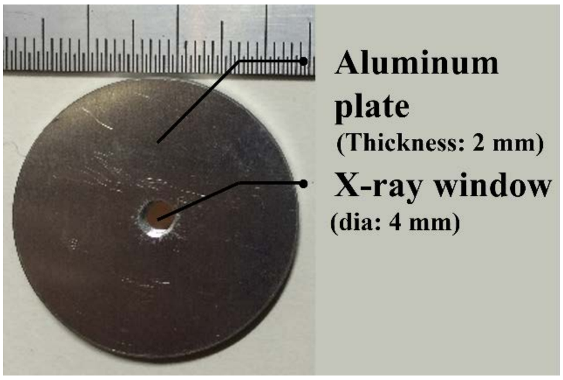

Figure S1.4. The aluminate alloy plate with an X-ray window for cover replacement

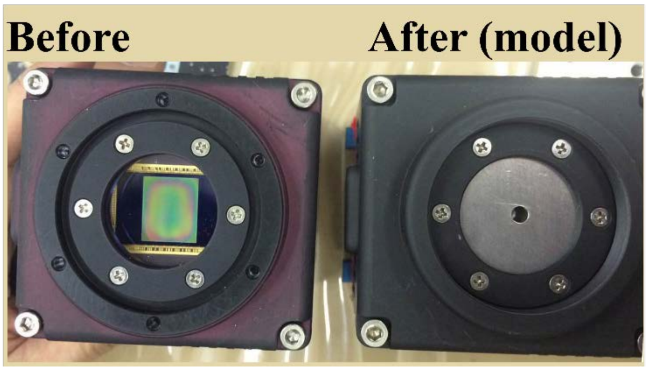

Figure S1.5. The direct comparison of the sCMOS camera before and after cover replacement

S2. Procedures of finding the empirical filter criteria

The experimental data used for this supplementary information is taken from a pellet sample containing MnO_2. The measurement condition is the same as that in the main text: the primary X-ray beam is monochromatic copper Kα; the single exposure time is set as 100 ms. In this experiment, the number of images is 1,000, hence the total accumulation time is 100 seconds.

Figure S2.1a shows the frequency distribution histogram of the integrated intensity of all photon events. It shows the preliminary X-ray fluorescence spectra before any filtering operation is adopted. It has a strong low energy background. Two regions of interest (ROI) in the spectra are defined: ROI 1 locates in the region of low energy background, the integrated intensity from 500 to 2,000; ROI 2 locates in the region of the Mn Kα peak, the integrated intensity from 2,500 to 2,650. The frequency distribution of the center-to-integrated ratio of photon events in two ROIs are surveyed and shown in Figure S2.1b and S2.1c, respectively. It is found that, the center-to-ratios of the photon events in ROI 1 are mostly below 40%, while those of some photon events in ROI 2 can exceed 40%. Therefore, the first criteria of 40% should be adopted to fully remove the low energy background noises.

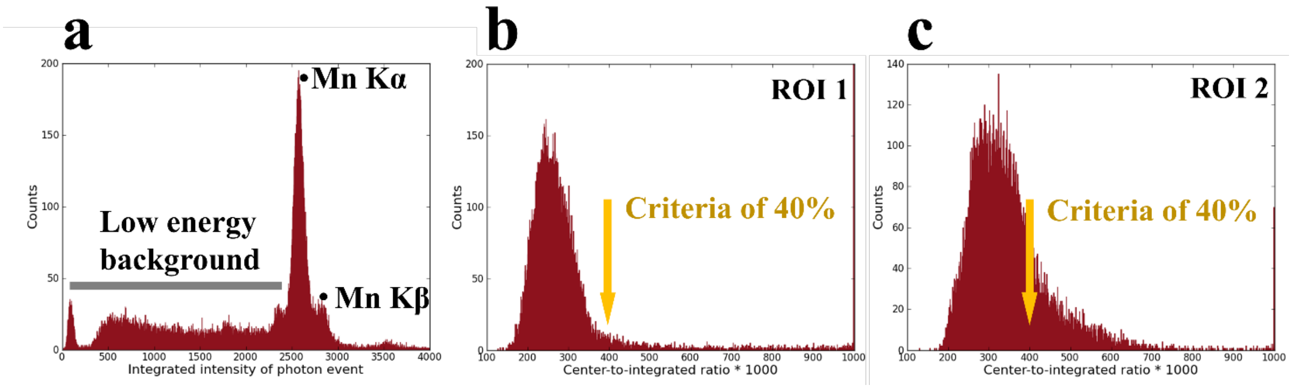

Figure S2.1 The preliminary X-ray fluorescence spectra before filtering

Figure S2.2a shows the X-ray fluorescence spectra after the first-time filtering. It only counts the photon events whose center-to-integrated ratio is larger than 40%. The Mn Kα peak is not symmetric. Its high energy extensions are stronger. In addition to this, there are two unknown noisy peaks in the spectra. This time, two ROIs in the spectra are defined: ROI 3 locates in the low energy park of the Mn Kα peak, the integrated intensity from 2,500 to 2,550; ROI 4 locates in the high energy part of the Mn Kα peak, the integrated intensity from 2,600 to 2,650. In a similar way, it is found that, the center-to-background ratios of photon events in ROI 3 (Figure S2.2b) are mostly below 50%, while those of some photon events in ROI 4 ((Figure S2.2c)) can exceed 50%. Therefore, the second criteria of 50% should be introduced to trim the Mn Kα peak to make it symmetric.

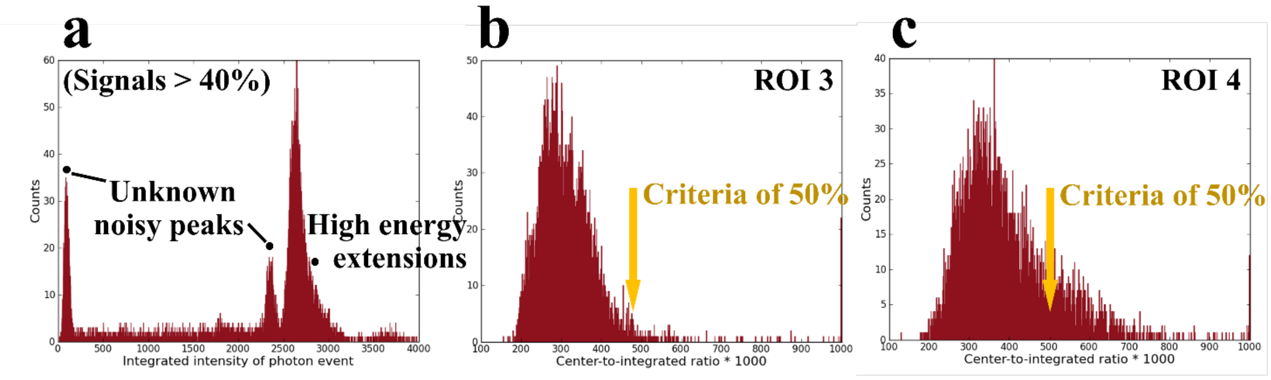

Figure S2.2 The X-ray fluorescence spectra after the first-time filtering

Figure S2.3a shows the final X-ray fluorescence spectra after twice filtering. It only counts the photon events whose center-to-integrated ratio is between 40% and 50%. As the frequency distribution of the center-to-integrated ratio of photon events in different ROIs are not distinctly different, many photon events have been filtered out. However, it is worthy to do this because the final spectra can exactly match the expected X-ray fluorescence spectra of the sample.

Figure S2.3b shows the spectra whose filtering criteria is [38%, 52%]. It is found that a small change to the filtering criteria will not make a big difference to the final spectra. However, we still prefer to use the criteria of [40%, 50%] to obtain the best spectra quality.

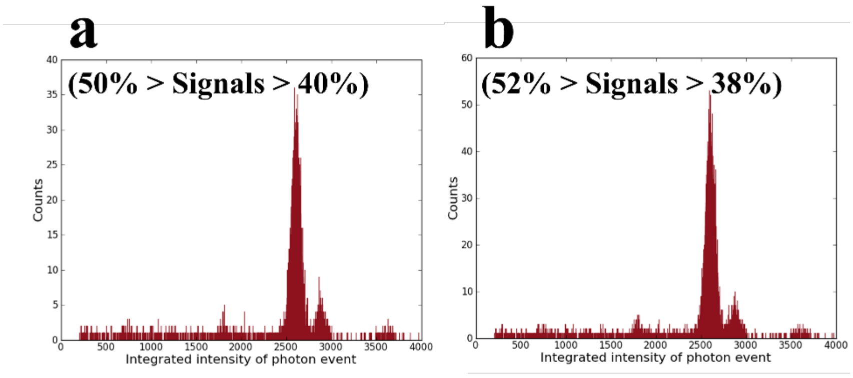

Figure S2.3 The final X-ray fluorescence spectra after twice filtering

This example shows that the empirical filtering criteria can be fluently found even if the experimental data is obtained in a very quick experiment (for example, 100 seconds). In our research, we have confirmed that this empirical filtering criteria is effective in all samples and experiment conditions which we have ever tested.
